# Supplementary material for: IRF4 deficiency vulnerates B-cell progeny for leukemogenesis via somatically acquired Jak3 mutations conferring IL-7 hypersensitivity
Source: Cell Death Differ. 2022 Apr 22;29(11):2163–76. doi: 10.1038/s41418-022-01005-z (PMC9613660; doi:10.1038/s41418-022-01005-z)
Supplement: Supplementary file 4 — supplementary information [file 41418_2022_1005_MOESM4_ESM.docx]

**IRF4 deficiency vulnerates B cell progeny for leukemogenesis via somatically acquired *Jak3* mutations conferring IL-7 hypersensitivity**

Running title: IRF4 deficiency vulnerates preB cells for leukemogenesis

Dennis Das Gupta^1^, Christoph Paul^2^, Nadine Samel^1,3^, Maria Bieringer^1^, Daniel Staudenraus^1^, Federico Marini^4^, Hartmann Raifer^1^, Lisa Menke^1^, Lea Hansal^1^, Bärbel Camara^1^, Edith Roth^5^, Patrick Daum^5^, Michael Wanzel^6^, Marco Mernberger^6,7^, Andrea Nist^6^, Uta-Maria Bauer^6^, Frederik Helmprobst^8,9^, Malte Buchholz^10^, Katrin Roth^11^, Lorenz Bastian^12^, Alina M Hartmann^12^, Claudia Baldus^12^, Koichi Ikuta^13^, Andreas Neubauer^14^, Andreas Burchert^14^, Hans-Martin Jäck^5^, Matthias Klein^15^, Tobias Bopp^15,16^, Thorsten Stiewe^6,7^, Axel Pagenstecher^8,9^, **Michael Lohoff^1*^**

^1^Institute for med. Microbiology & Hospital Hygiene, Philipps University Marburg, Germany

^2^University Hospital Gießen and Marburg, and Philipps University, Dept. Ophthalmology, *Marburg, Germany*

3MVZ for Laboratory Medicine and Microbiology, Koblenz-Mittelrhein, Germany

^4^Institute of Medical Biostatistics, Epidemiology and Informatics (IMBEI), University Medical Center of the Johannes Gutenberg-University Mainz, Germany

*^5^*Division of Molecular Immunology, Nikolaus-Fiebiger Center, University of Erlangen-Nürnberg, Erlangen, Germany

^6^Institute for Molecular Biology and Tumor Research (IMT), Center for Tumor- and Immunobiology (ZTI), Philipps University Marburg, Germany

^7^Genomics Core Facility, Philipps University Marburg, Germany

^8^Core Facility for Mouse Pathology and Electron Microscopy, Philipps University Marburg, Germany

^9^University Hospital Gießen and Marburg, and Philipps University, Institute of Neuropathology, Marburg, Germany

*^10^*University Hospital Gießen and Marburg, and Philipps University, Clinic for Gastroenterology and Core Facility Small Animal Ultrasound, Marburg, Germany

^11^Core facility for Cellular Imaging, Philipps University Marburg, Germany

^12^Medical Department II, Hematology and Oncology, University Medical Center Schleswig- Holstein, Kiel, Germany

^13^Institute for Frontier Life and Medical Sciences, Kyoto University, Japan

*^14^*University Hospital Gießen and Marburg, and Philipps University, Dept. Hematology, Oncology and Immunology, *Marburg,* Germany

^15^Institute for Immunology, Research Center for Immunotherapy (FZI), University Cancer Center, University Medical Center of the Johannes Gutenberg-University Mainz, Germany

^16^German Cancer Consortium (DKTK)

***corresponding author: lohoff@med.uni-marburg.de**


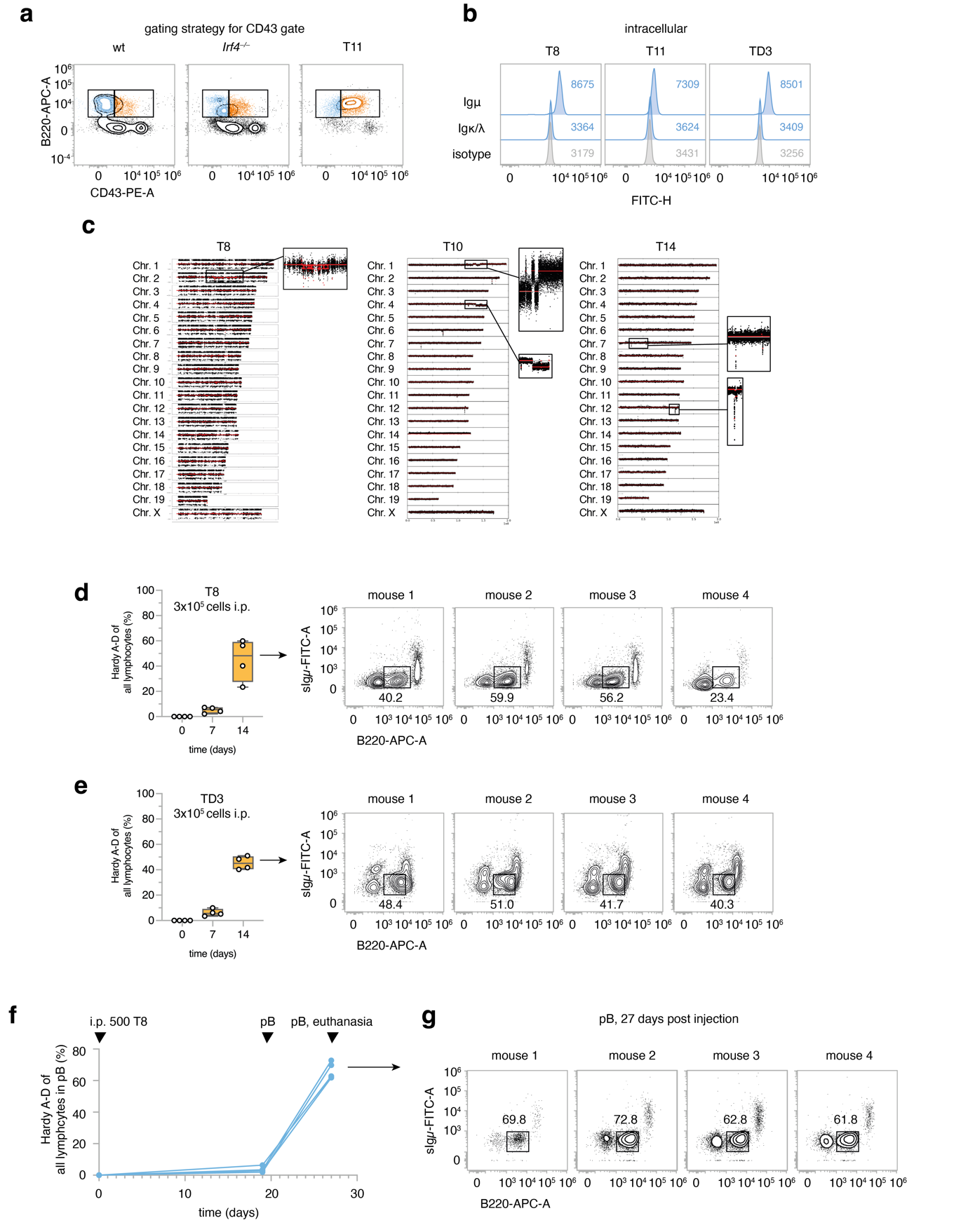


**Supplementary Fig.1: extended data to Fig.1**

**a** gating strategy for Fig.1j: wt and *Irf4^–/–^* control BM stainings were used to identify the CD43^+^ cell gate used for tumour cell phenotyping **b** T8, T11 and TD3 were stained for intracellular Igμ and light chain (Igκ/λ) expression or isotype control. Numbers indicate geometric mean fluorescence intensity. **c** gDNA (T10, T14) or exome (T8) libraries were sequenced and analyzed using the cn.mops pipeline for copy number variations (CNV); inserts highlight altered chromosomal regions. **d-e** four wt mice were *i*.*p*. injected with 5x10^3^ T8 (**d**) or TD3 (**e**) cells and occurrence of B220^+^sIgµ- cells in the peripheral blood was assessed at day 7 and day 14 post injection. Boxes represent mean and 95 % confidence intervals, whiskers minimum and maximum. Right panels show data at day 14 post injection of the respective four individual mice. **f** four wt mice were *i*.*p*. injected with 500 T8 cells. After 19- and 27-days tail vein blood (pB) was analyzed by flow cytometry for the presence of B220^mid^ Igμ^–^ leukemia cells. Dots represent individual mice. **g** flow cytometric analysis of pB for of B220 and sIgμ at 27 days post injection. Numbers indicate frequency within the respective gate (%).

**
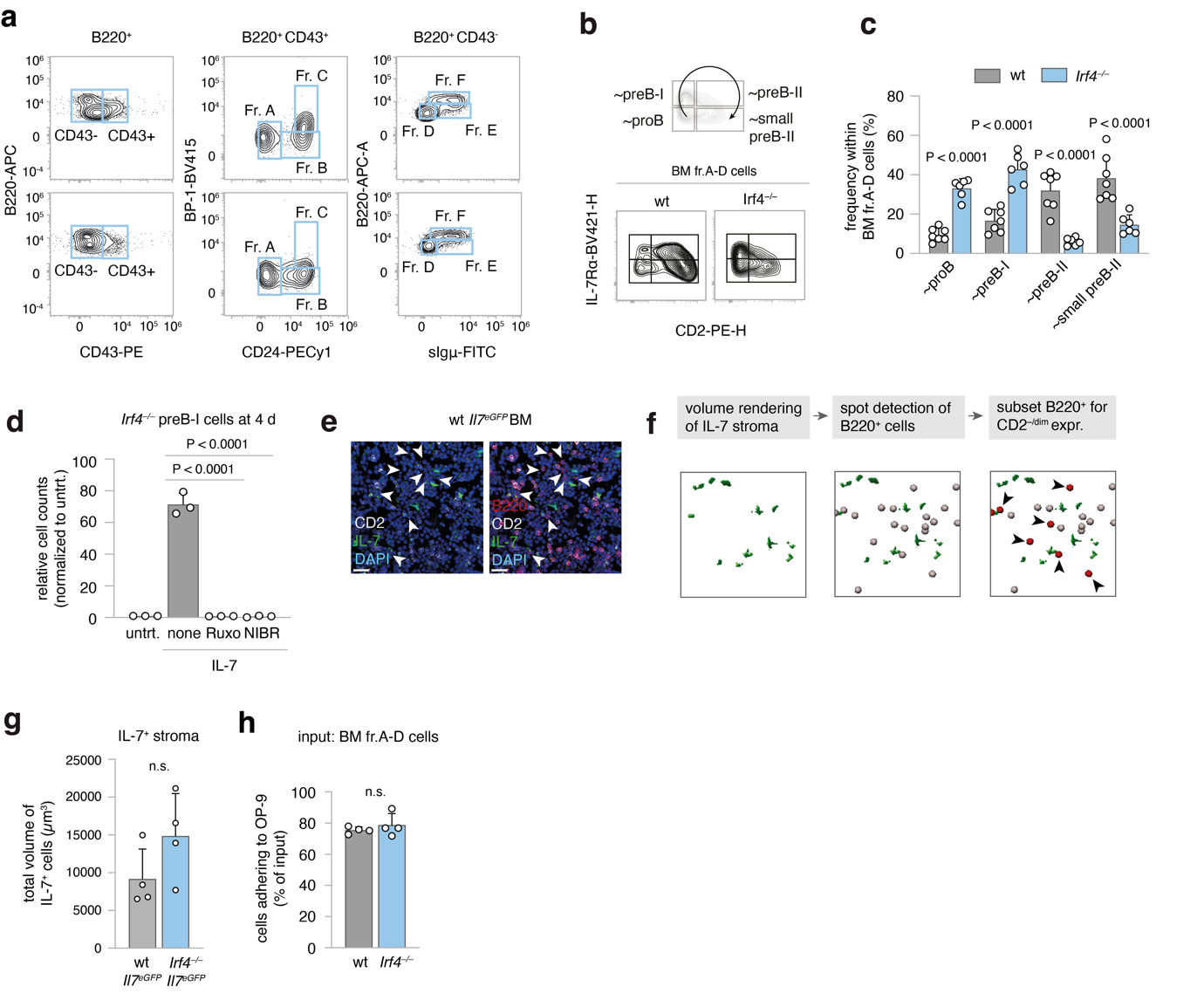
**

**Supplementary Fig.2: extended data to Fig.2**

**a** Gating strategy for Hardy fragment analysis in Fig.2a-f. Gated B220^+^, B220^+^CD43^+^ and B220^+^CD43^-^ were analyzed for expression of the indicated markers. **b** within the fr.A-D cell gate, BM cells were analyzed for CD2 and IL-7Rα expression. Top pictogram presenting developmental path within the gated quadrants. **c** Quantification of cell frequency within the fr.A-D cell gate (**b**) for n = 7 (wt) and n = 6 (*Irf4^–/–^*) mice. Analyzed with two-way ANOVA, Sidak post-hoc **d** *Irf4^–/–^* BM cells were cultured for 4 days in the presence of the indicated substances and cell counts recorded, presented as normalized to untrt. = untreated. Ruxo = Ruxolitinib, NIBR = NIBR3049. One-way ANOVA, Tukey post-hoc **e** Overlay confocal microscopic images of wt *il7^eGFP^* femur cryosections. Arrowheads indicate B220^+^CD2^–/dim^ cells. Scale bars = 20 µm **f** strategy for comparing B cell progenitor proximity to IL-7^+^ BMSCs in BM cryosections using the IMARIS software (see methods). Briefly, GFP^+^ signal was rendered as volumes, B220^+^ cells were detected as spots and subsetted (red) for CD2^–/dim^ expression. Distances were measured from each spot to the nearest GFP^+^ volume surface. Arrowheads = B220^+^, CD2^–/dim^ cells. See also supplementary movie 1. **g** total volume of GFP^+^ cellular structures was measured for each cryosection (n = 4 per genotype). Each dot represents one cryosection from one biological replicate. Unpaired two-tailed t-test. **h** fr.A-D cells were MACS purified from BM and seeded onto monolayers of OP-9 cells. Non-adhering cells were counted after 1 hours. Adherent cell counts calculated as n_adherent =_ n_input_ – n_non-adherent_. Data depicts n_adherent_ / n_input_ as percentages. Unpaired two-tailed t-test. Bars represent mean ± SD in all panels.


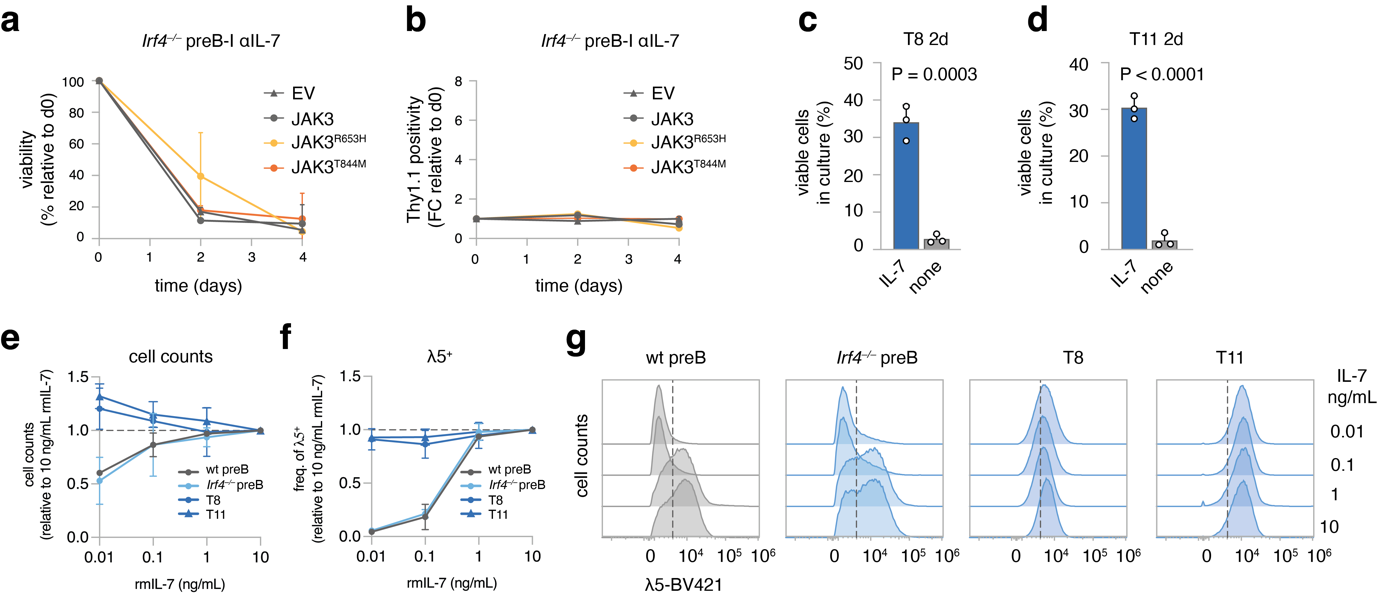


**Supplementary Fig.3: extended data to Fig.3 å**

**a** *Irf4^–/–^* preB-I cell cultures from BM cells were transduced with RV as in Fig.2e-g and seeded in the presence of anti(α)-IL-7. Viability of cells is plotted over the course of 4 days relative to viability at day 0. **b** Thy1.1^+^ cell frequency relative to day 0 is plotted for cells as in (**a**). Data as mean ± SD of n = 3 independent experiments for (**a-b**). **c** T8 and **d** T11 cells were cultured for 48 h in the presence or absence of 10 ng/mL IL-7 and viability of culture was recorded. Dots indicate n = 3 independent experiments summarized as bars (mean ± SD), two-tailed unpaired t-test. **e-g** wt and *Irf4^–/–^* preB cell cultures were generated by culturing BM cells for six days in the presence of 10 ng/mL IL-7. The ability of preB cells to further proliferate and maintain preB cell identity in decreasing concentrations of IL-7 was then assessed in comparison to T8 and T11 leukemia cells over 48 h. **e** cell counts and **f-g** lambda5 surface expression were measured flow cytometrically. The dashed line in **g** marks positive staining. Three independent experiments, data in **e-f** as mean ± SD.

**
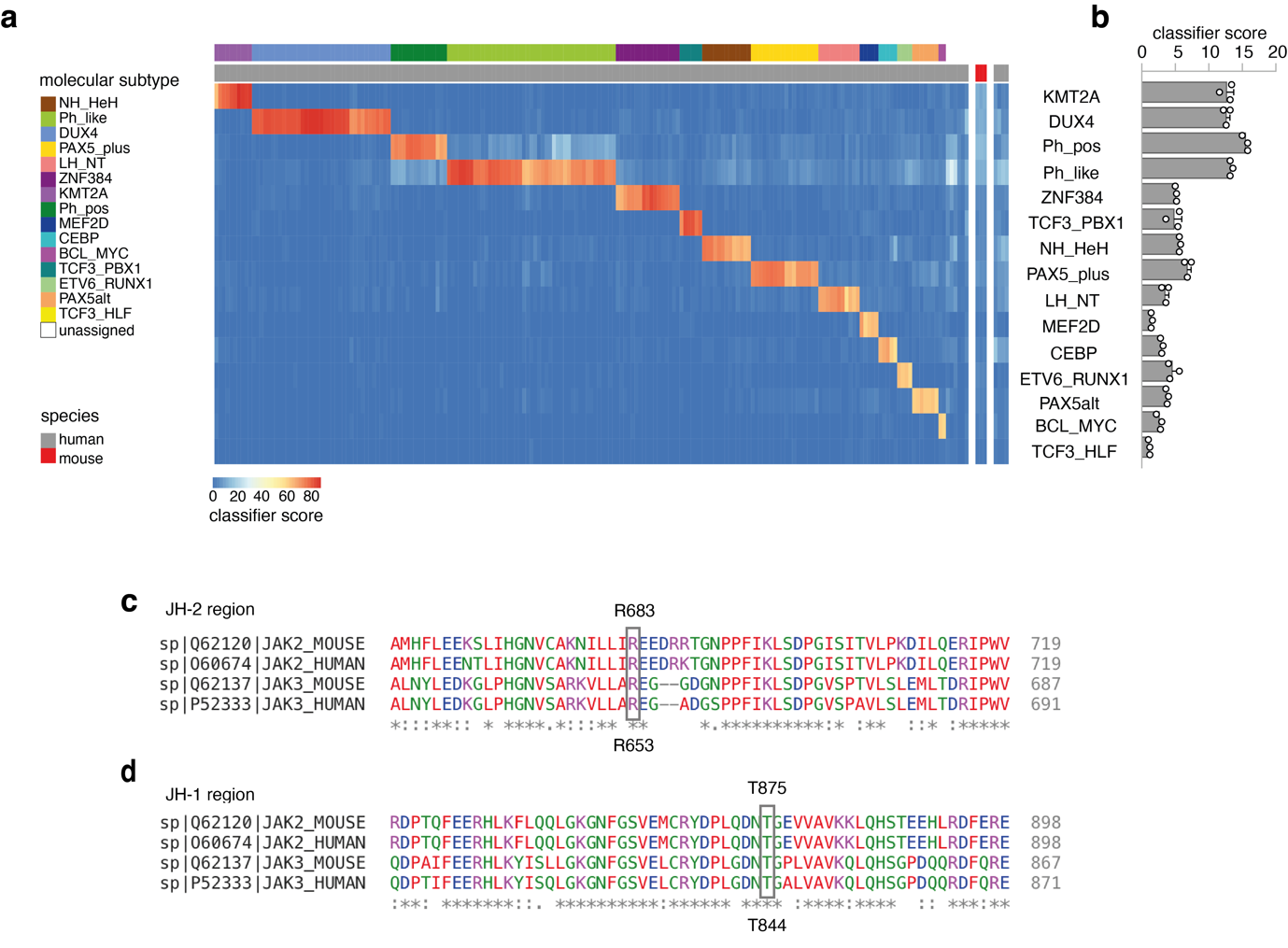
**

**Supplementary Fig.4: extended data to Fig.4**

**a** expression of human-mouse orthologue genes was compared between a published BCP-ALL patient cohort and *Irf4^–/–^* leukemia samples. A random forest classifier trained on the human samples was used to generate BCP-ALL subtype prediction scores for *Irf4^–/–^* leukemia samples. Heatmap presents individual patient/mouse samples as columns, ALL subtypes as rows, colour-fills give prediction scores per row. Colour labels above heatmap indicate the molecular BCP-ALL subtype of each human sample (first row) and the species (human/mouse, second row) as explained to the left of the heatmap. *Irf4^–/–^* leukemia samples are separated from human samples by white space. **b** classifier scores for *Irf4^–/–^* leukemia are plotted adjacent to the corresponding row in (**a**) for the n = 3 tested tumours, indicated by dots, summarized by bars (mean ± SD) **c-d** Clustal omega sequence alignment of mouse and human JAK2 and JAK3 **c** JH2 and **d** JH1 loop regions in mouse and human. R683/R653 and T875/T844 amino acids are highlighted.


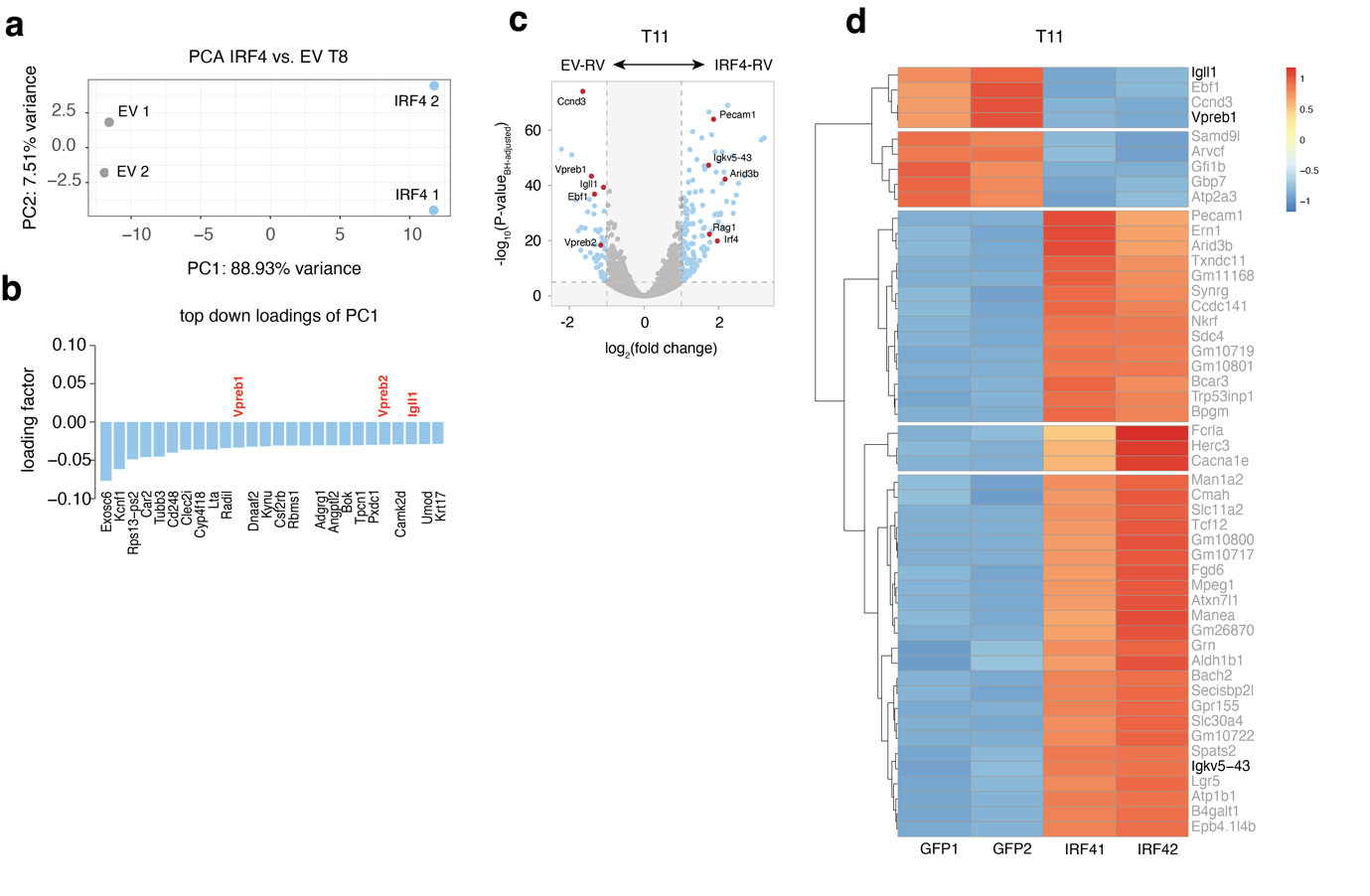


**Supplementary Fig.5: extended data to Fig.5**

**a** Principal components (PC) 1 and 2 are presented for expression data from Fig.5d-h. **b** top down-pointing loadings for PC1 are presented and ψL components highlighted. **c-d** T11 cells transduced with RVs as described in Fig.5. Total RNA sequenced 24 h after transduction. **c** Volcano plot: log_2_ of fold change between conditions (IRF4-RV vs. EV-RV) against respective -log_10_ of Benjamini-Hochberg adjusted p-values. ψL components and the differentiation genes *Igkv*, *Rag1* and *Irf4* are highlighted. Dotted lines mark x- and y- cutoff values. **d** Heatmap depicting the 50 most significantly regulated genes. Euclidean clustering depicted as dendrograms to the left. Two samples per group.

**
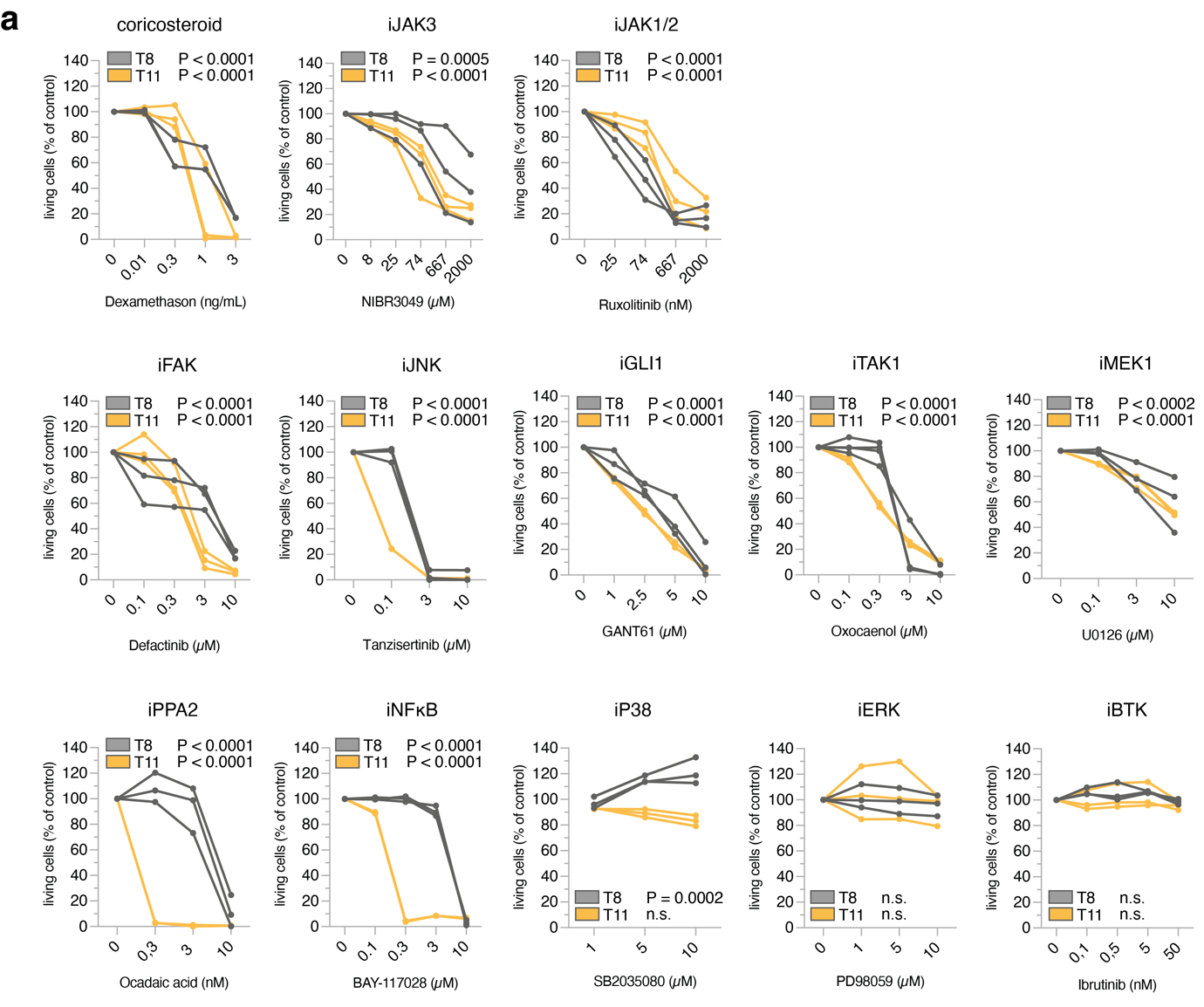
**

**Supplementary Fig.6: small compound inhibitors targeting leukemia cell survival**

**a** 2.5x10^5^ T8.1 or T11 cells per well were cultured in a 48 well plate in the presence of the indicated concentrations of inhibitors listed below the x-axis. The inhibitors target the pathways described above of the panels (exception: dexamethasone). To determine the percentage of viable cells, samples were stained using Annexin V and propidium/iodide after 48 h. All values are relative to initial viability at the onset of the experiment which was arbitrarily set to 100 %. Results give the mean ± SD of at least three separate experiments per inhibitor. Unpaired two-tailed t-tests comparing viability at highest to lowest concentration of inihibitor.

**
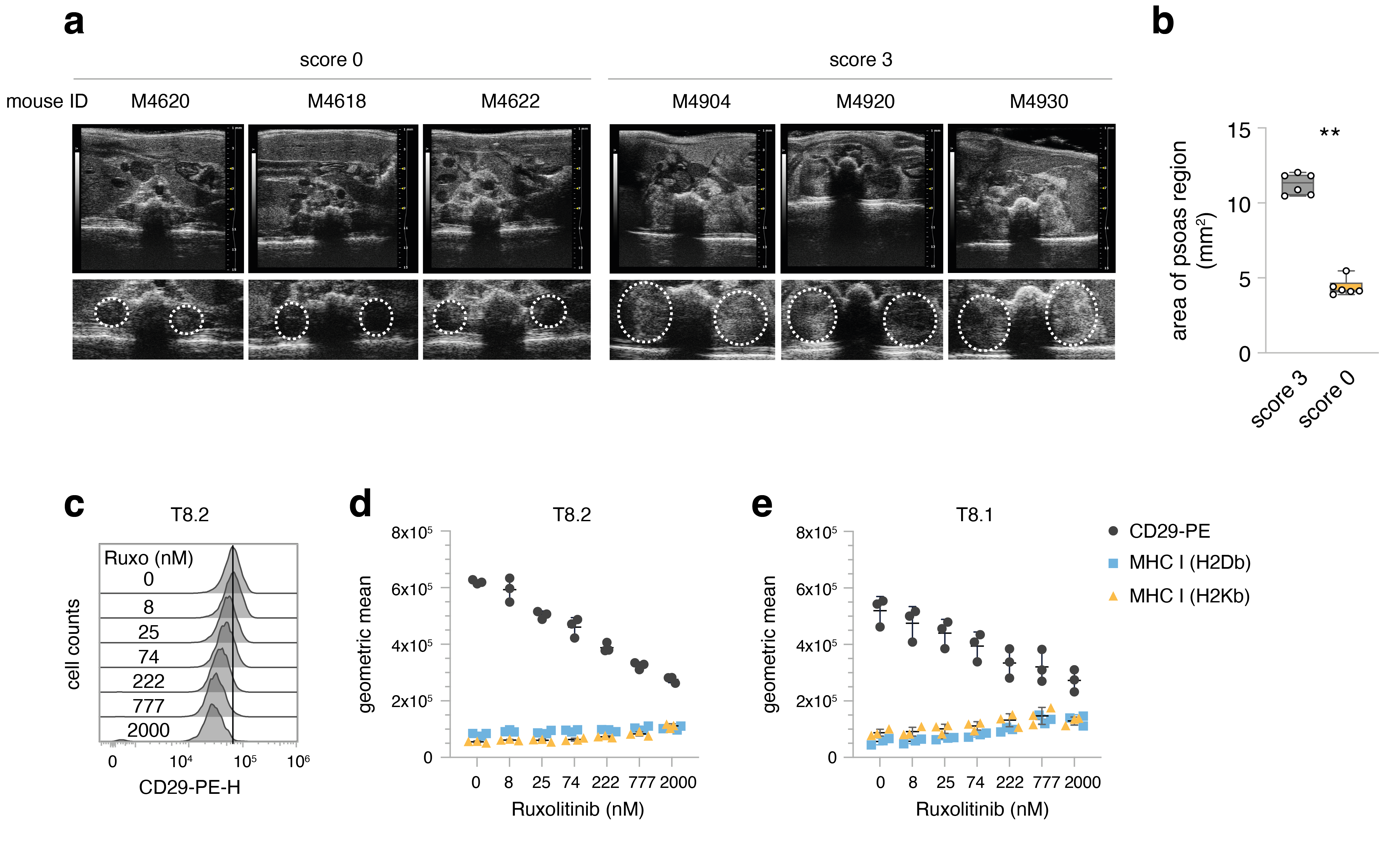
**

**Supplementary Fig.7: extended data to Fig.6**

**a** Ultrasound scan of the paravertebral lumbar area of individual (see mouse ID) tumour-affected mice. The region of the psoas muscle is highlighted as a circle, the area of which is measured. **b** Bars show the mean area ± SD of the psoas region for three mice with score 0 and 3, analyzed by two-tailed unpaired t-test. **c-e** T8.1 and T8.2 cells were cultured for 48 h in the presence of varying concentrations of Ruxolitinib. Thereafter, CD29, MHC I (H2Db and H2Kb) surface expression were measured. **c** Representative histogram for CD29 expression on T8.2 exposed to Ruxolitinib. **d** Quantification of results for T8.2, **e** Quantification for T8.1. Two-Way ANOVA, Sidak post-hoc comparisons to data for 0 nM Ruxolitinib.

**
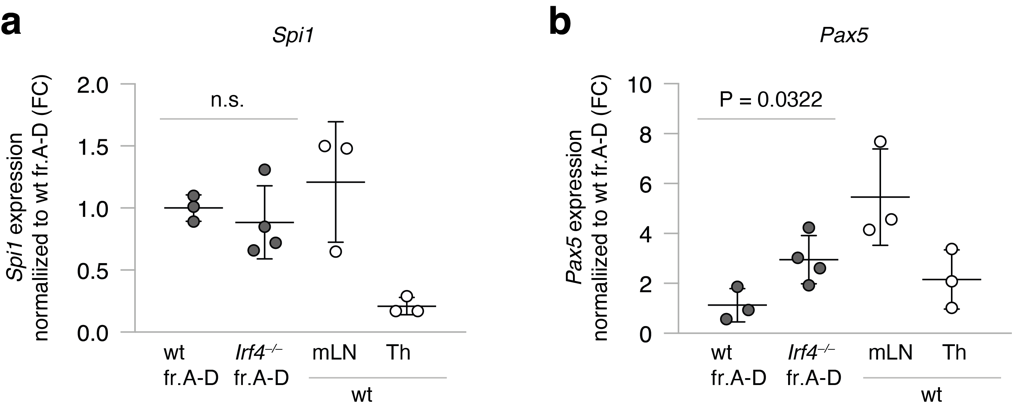
**

**supplementary Fig.8: *Pax5* and *Spi1* are not downregulated in *Irf4^–/–^* fr.A-D cells**

**a** *Spi1* and **b** *Pax5* gene expression in sorted *Irf4^–/–^* and wt fr.A-D cells, as well as wt mLN and T helper (Th) control samples. n = 3 biological replicates for all but *Irf4^–/–^* fr.A-D cells (n=4). Mean ± SD, dots represent individual samples, Two-tailed unpaired t-test comparing wt to *Irf4^–/–^* fr.A-D cells
