# Supplementary material for: IRF4 deficiency vulnerates B-cell progeny for leukemogenesis via somatically acquired Jak3 mutations conferring IL-7 hypersensitivity
Source: Cell Death Differ. 2022 Apr 22;29(11):2163–76. doi: 10.1038/s41418-022-01005-z (PMC9613660; doi:10.1038/s41418-022-01005-z)
Supplement: Supplementary file 7 — Author contribution form 2/3 [file 41418_2022_1005_MOESM7_ESM.pdf]

DECLARATION OF CONTRIBUTIONS TO ARTICLE

ADMC

Manuscript Number: CDD-22-0273R

Journal Name: *Cell Death & Differentiation* (the 'Journal')

Proposed Title of the Contribution:

IRF4 deficiency vulnerates B cell progeny for leukemogenesis via somatically acquired Jak3 mutations conferring IL-7 hypersensitivity (the 'Contribution)

Author(s):

Mr Dennis Das Gupta , Dr Christoph Paul , Dr Nadine Samel , Dr Maria Bieringer , Mr Daniel Staudenraus , Dr Federico Marini , Dr Hartmann Raifer , Dr Lisa Menke , Ms Lea Hansal , Ms Bärbel Camara , Dr Edith Roth , Dr Patrick Daum , Dr Michael Wanzel , Dr Marco Mernberger , Dr Andrea Nist , Professor Uta-Maria Bauer , Dr Frederik Helprobst , Malte Buchholz , Dr Katrin Roth , Dr Lorenz Bastian , Dr Alina Hartmann , Professor Claudia Baldus , Professor Koichi Ikuta , Andreas Neubauer , Professor Andreas Burchert , Professor Hans-Martin Jäck , Dr Matthias Klein , Professor Tobias Bopp , Professor Thorsten Stiewe , Professor Axel Pagenstecher (the 'Authors')

For all *CDD* articles, each person named as an author in the published version must be able to show he or she has contributed substantially to the article.

Authorship credit should be based on 1) substantial contributions to conception and design, acquisition of data, or analysis and interpretation of data; 2) drafting the article or revising it critically for important intellectual content; and 3) final approval of the version to be published. Authors should meet conditions 1, 2 and 3.

Any person who cannot be shown to have made a substantial contribution to the article cannot be listed as an author in the final version. The name of any person who is deemed to have made a minor contribution can, however, appear in the Acknowledgments section of the article.

Please complete the table below to indicate the contributions of all named authors to the manuscript.

Author Full Name:	Specification of Contribution to the Manuscript:
Marco Mernberger	analysis, interpretation, approval
Andrea Nist	acquisition, analysis, approval
Uta-Maria Bauer	acquisition, analysis, interpretation, approval
Frederik Helmprobst	acquisition, analysis, interpretation, approval
Malte Buchholz	acquistion, analysis, approval
Katrin Roth	acquistion, analysis, interpretation, approval
Lorenz Bastian	analysis, interpretation, approval
Alina Hartmann	analysis, interpretation, approval
Claudia Baldus	interpretation, revising, approval
Koichi Ikuta	analysis, acquisition, approval
Andreas Neubauer	interpretation, revising, approval
Andreas Burchert	interpretation, revising, approval
Hans-Martin Jäck	interpretation, revising, approval

Please complete the table below to indicate the contributions of all named authors to the figures.

Figure 1:

AN, AB, HMJ

Figure 2:

KR, KI

Figure 3:

MM, AN, UMB

Figure 4:

LB, AH, CB

Figure 5:

Figure 6:

FH, MB

Signed for and on behalf of the Author(s):	Print Name:	Date:
	Dennis Das Gupta	2022-03-31
